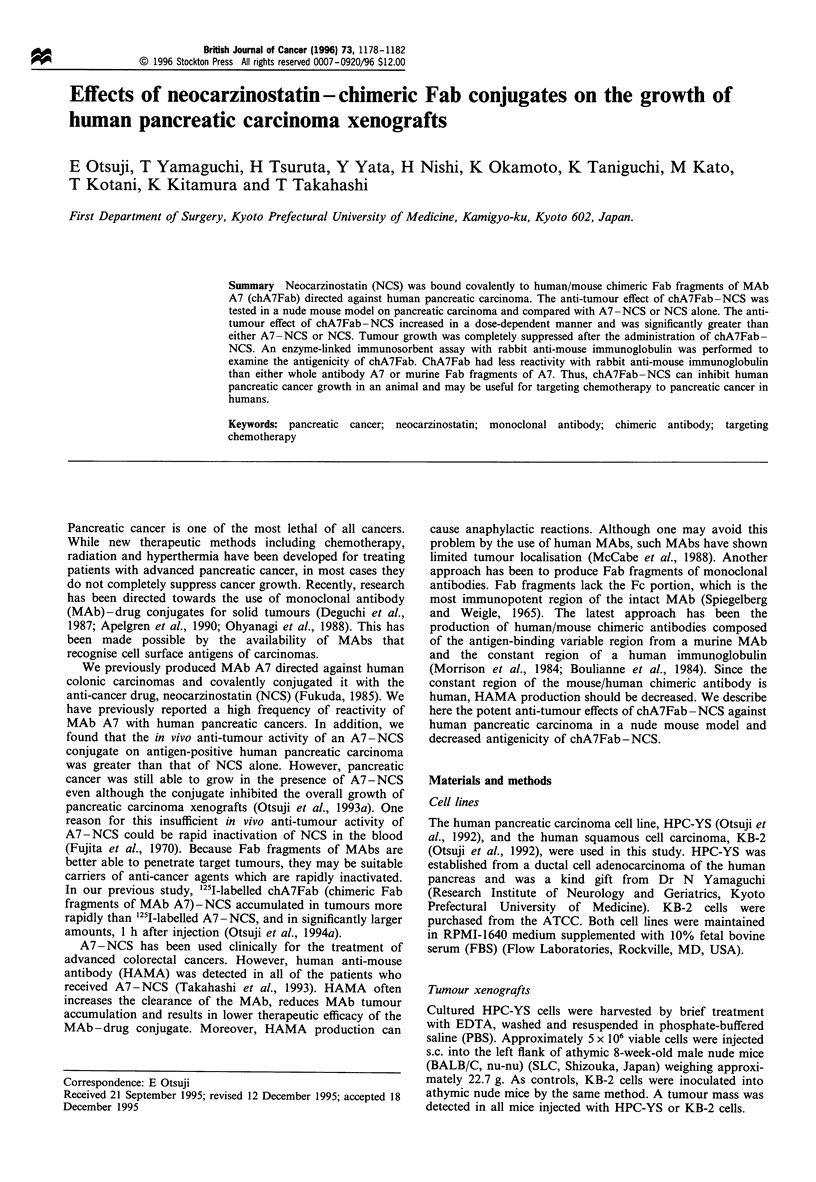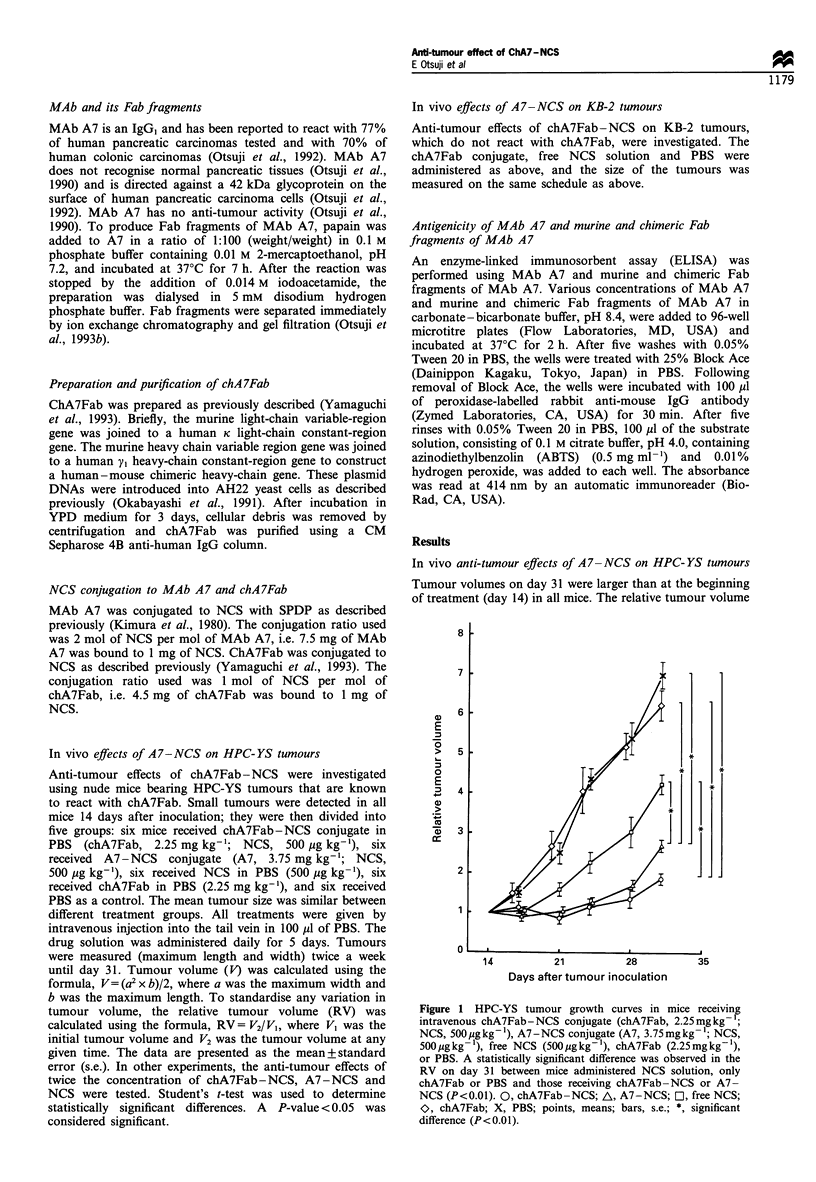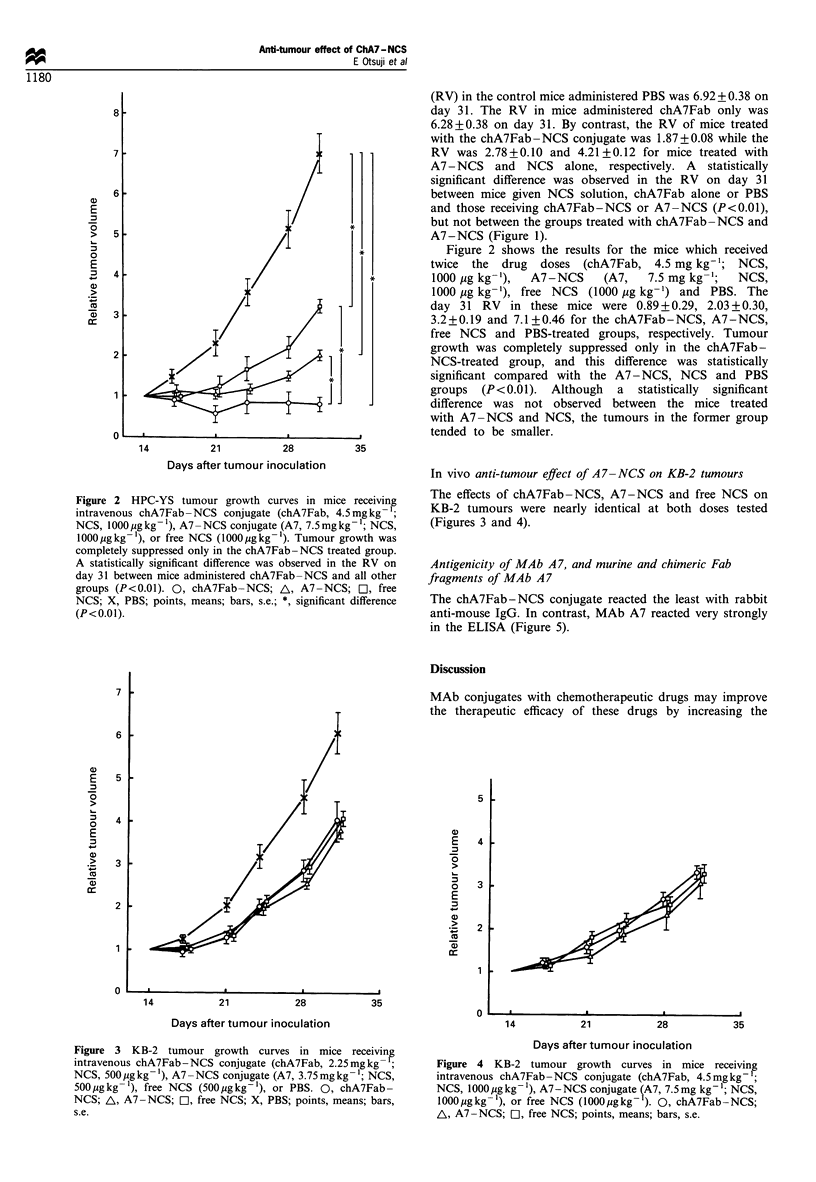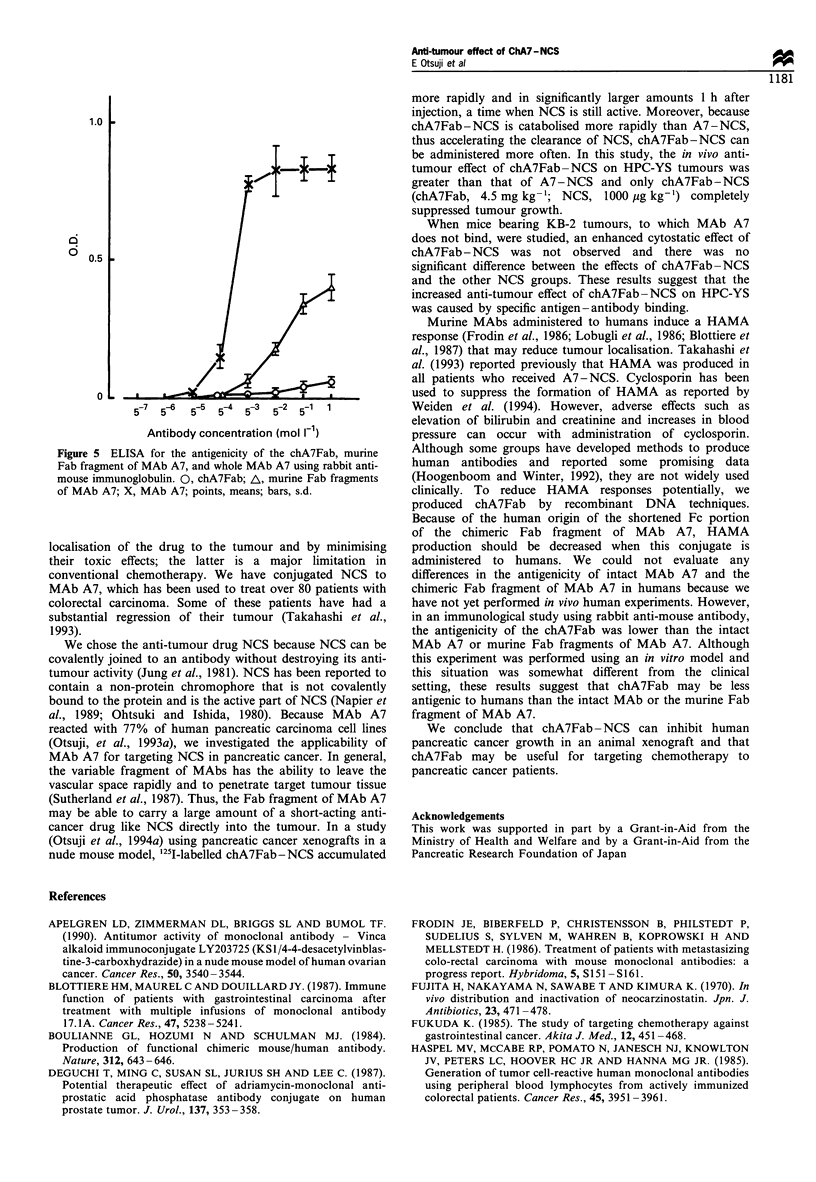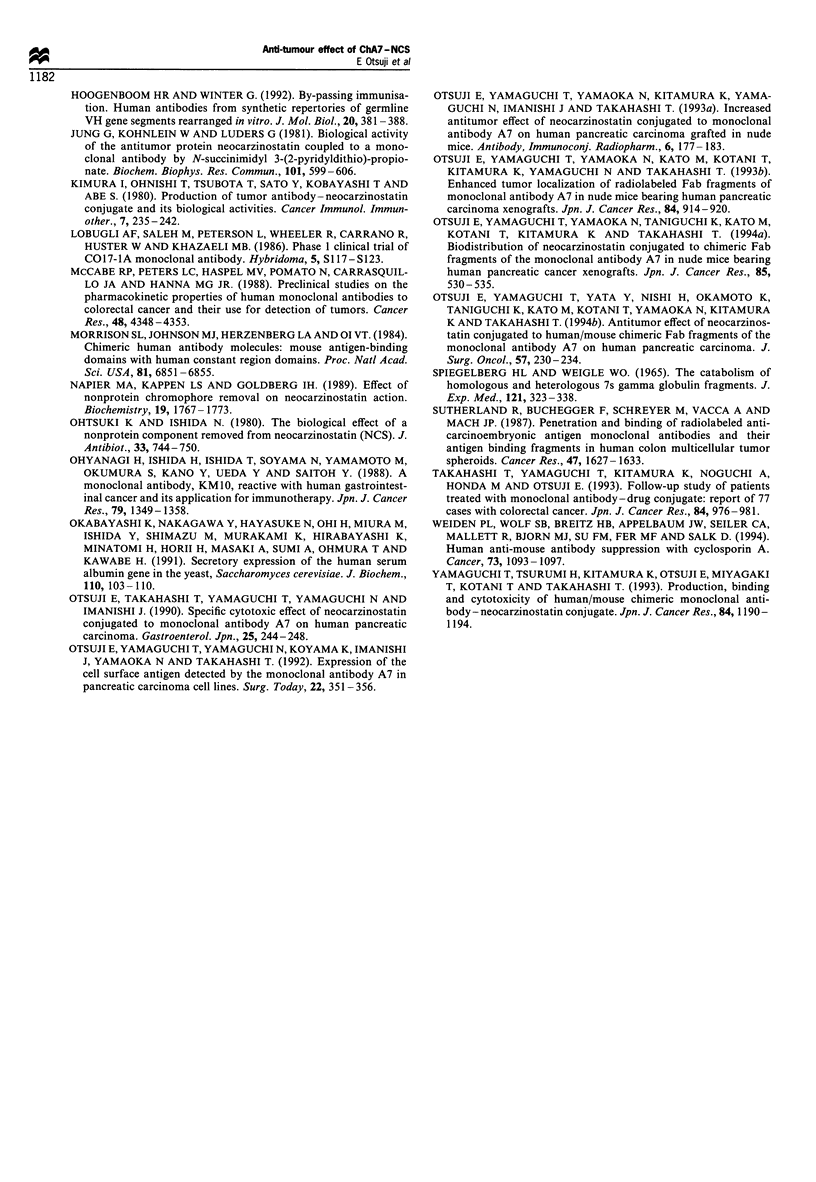# Effects of neocarzinostatin-chimeric Fab conjugates on the growth of human pancreatic carcinoma xenografts.

**DOI:** 10.1038/bjc.1996.227

**Published:** 1996-05

**Authors:** E. Otsuji, T. Yamaguchi, H. Tsuruta, Y. Yata, H. Nishi, K. Okamoto, K. Taniguchi, M. Kato, T. Kotani, K. Kitamura, T. Takahashi

**Affiliations:** First Department of Surgery, Kyoto Prefectural University of Medicine, Japan.

## Abstract

Neocarzinostatin (NCS) was bound covalently to human/mouse chimeric Fab fragments of MAb A7 (chA7Fab) directed against human pancreatic carcinoma. The anti-tumour effect of chA7Fab-NCS was tested in a nude mouse model on pancreatic carcinoma and compared with A7-NCS or NCS alone. The anti-tumour effect of chA7Fab-NCS increased in a dose-dependent manner and was significantly greater than either A7-NCS or NCS. Tumour growth was completely suppressed after the administration of chA7Fab-NCS. An enzyme-linked immunosorbent assay with rabbit anti-mouse immunoglobulin was performed to examine the antigenicity of chA7Fab. ChA7Fab had less reactivity with rabbit anti-mouse immunoglobulin than either whole antibody A7 or murine Fab fragments of A7. Thus, chA7Fab-NCS can inhibit human pancreatic cancer growth in an animal and may be useful for targeting chemotherapy to pancreatic cancer in humans.


British Journal of Cancer (1996) 73, 1178-1182
© 1996 Stockton Press All rights reserved 0007-0920/96 $12.00

### Effects of neocarzinostatin–chimeric Fab conjugates on the growth of human pancreatic carcinoma xenografts

E Otsuji, T Yamaguchi, H Tsuruta, Y Yata, H Nishi, K Okamoto, K Taniguchi, M Kato, T Kotani, K Kitamura and T Takahashi

*First Department of Surgery, Kyoto Prefectural University of Medicine, Kamigyo-ku, Kyoto 602, Japan.*

**Summary** Neocarzinostatin (NCS) was bound covalently to human/mouse chimeric Fab fragments of MAb A7 (chA7Fab) directed against human pancreatic carcinoma. The anti-tumour effect of chA7Fab–NCS was tested in a nude mouse model on pancreatic carcinoma and compared with A7–NCS or NCS alone. The anti-tumour effect of chA7Fab–NCS increased in a dose-dependent manner and was significantly greater than either A7–NCS or NCS. Tumour growth was completely suppressed after the administration of chA7Fab–NCS. An enzyme-linked immunosorbent assay with rabbit anti-mouse immunoglobulin was performed to examine the antigenicity of chA7Fab. ChA7Fab had less reactivity with rabbit anti-mouse immunoglobulin than either whole antibody A7 or murine Fab fragments of A7. Thus, chA7Fab–NCS can inhibit human pancreatic cancer growth in an animal and may be useful for targeting chemotherapy to pancreatic cancer in humans.

**Keywords:** pancreatic cancer; neocarzinostatin; monoclonal antibody; chimeric antibody; targeting chemotherapy

Pancreatic cancer is one of the most lethal of all cancers. While new therapeutic methods including chemotherapy, radiation and hyperthermia have been developed for treating patients with advanced pancreatic cancer, in most cases they do not completely suppress cancer growth. Recently, research has been directed towards the use of monoclonal antibody (MAb)–drug conjugates for solid tumours (Deguchi *et al.*, 1987; Apelgren *et al.*, 1990; Ohyanagi *et al.*, 1988). This has been made possible by the availability of MAbs that recognise cell surface antigens of carcinomas.

We previously produced MAb A7 directed against human colonic carcinomas and covalently conjugated it with the anti-cancer drug, neocarzinostatin (NCS) (Fukuda, 1985). We have previously reported a high frequency of reactivity of MAb A7 with human pancreatic cancers. In addition, we found that the *in vivo* anti-tumour activity of an A7–NCS conjugate on antigen-positive human pancreatic carcinoma was greater than that of NCS alone. However, pancreatic cancer was still able to grow in the presence of A7–NCS even although the conjugate inhibited the overall growth of pancreatic carcinoma xenografts (Otsuji *et al.*, 1993*a*). One reason for this insufficient *in vivo* anti-tumour activity of A7–NCS could be rapid inactivation of NCS in the blood (Fujita *et al.*, 1970). Because Fab fragments of MAbs are better able to penetrate target tumours, they may be suitable carriers of anti-cancer agents which are rapidly inactivated. In our previous study, $^{125}$I-labelled chA7Fab (chimeric Fab fragments of MAb A7)–NCS accumulated in tumours more rapidly than $^{125}$I-labelled A7–NCS, and in significantly larger amounts, 1 h after injection (Otsuji *et al.*, 1994*a*).

A7–NCS has been used clinically for the treatment of advanced colorectal cancers. However, human anti-mouse antibody (HAMA) was detected in all of the patients who received A7–NCS (Takahashi *et al.*, 1993). HAMA often increases the clearance of the MAb, reduces MAb tumour accumulation and results in lower therapeutic efficacy of the MAb–drug conjugate. Moreover, HAMA production can cause anaphylactic reactions. Although one may avoid this problem by the use of human MAbs, such MAbs have shown limited tumour localisation (McCabe *et al.*, 1988). Another approach has been to produce Fab fragments of monoclonal antibodies. Fab fragments lack the Fc portion, which is the most immunopotent region of the intact MAb (Spiegelberg and Weigle, 1965). The latest approach has been the production of human/mouse chimeric antibodies composed of the antigen-binding variable region from a murine MAb and the constant region of a human immunoglobulin (Morrison *et al.*, 1984; Boulianne *et al.*, 1984). Since the constant region of the mouse/human chimeric antibody is human, HAMA production should be decreased. We describe here the potent anti-tumour effects of chA7Fab–NCS against human pancreatic carcinoma in a nude mouse model and decreased antigenicity of chA7Fab–NCS.

### Materials and methods

### *Cell lines*

The human pancreatic carcinoma cell line, HPC-YS (Otsuji *et al.*, 1992), and the human squamous cell carcinoma, KB-2 (Otsuji *et al.*, 1992), were used in this study. HPC-YS was established from a ductal cell adenocarcinoma of the human pancreas and was a kind gift from Dr N Yamaguchi (Research Institute of Neurology and Geriatrics, Kyoto Prefectural University of Medicine). KB-2 cells were purchased from the ATCC. Both cell lines were maintained in RPMI-1640 medium supplemented with 10% fetal bovine serum (FBS) (Flow Laboratories, Rockville, MD, USA).

### *Tumour xenografts*

Cultured HPC-YS cells were harvested by brief treatment with EDTA, washed and resuspended in phosphate-buffered saline (PBS). Approximately $5 \times 10^6$ viable cells were injected s.c. into the left flank of athymic 8-week-old male nude mice (BALB/C, nu-nu) (SLC, Shizouka, Japan) weighing approximately 22.7 g. As controls, KB-2 cells were inoculated into athymic nude mice by the same method. A tumour mass was detected in all mice injected with HPC-YS or KB-2 cells.

Correspondence: E Otsuji
Received 21 September 1995; revised 12 December 1995; accepted 18 December 1995

*MAb and its Fab fragments*

MAb A7 is an $IgG_1$ and has been reported to react with 77% of human pancreatic carcinomas tested and with 70% of human colonic carcinomas (Otsuji *et al.*, 1992). MAb A7 does not recognise normal pancreatic tissues (Otsuji *et al.*, 1990) and is directed against a 42 kDa glycoprotein on the surface of human pancreatic carcinoma cells (Otsuji *et al.*, 1992). MAb A7 has no anti-tumour activity (Otsuji *et al.*, 1990). To produce Fab fragments of MAb A7, papain was added to A7 in a ratio of 1:100 (weight/weight) in 0.1 M phosphate buffer containing 0.01 M 2-mercaptoethanol, pH 7.2, and incubated at 37°C for 7 h. After the reaction was stopped by the addition of 0.014 M iodoacetamide, the preparation was dialysed in 5 mM disodium hydrogen phosphate buffer. Fab fragments were separated immediately by ion exchange chromatography and gel filtration (Otsuji *et al.*, 1993*b*).

*Preparation and purification of chA7Fab*

ChA7Fab was prepared as previously described (Yamaguchi *et al.*, 1993). Briefly, the murine light-chain variable-region gene was joined to a human $\kappa$ light-chain constant-region gene. The murine heavy chain variable region gene was joined to a human $\gamma_1$ heavy-chain constant-region gene to construct a human-mouse chimeric heavy-chain gene. These plasmid DNAs were introduced into AH22 yeast cells as described previously (Okabayashi *et al.*, 1991). After incubation in YPD medium for 3 days, cellular debris was removed by centrifugation and chA7Fab was purified using a CM Sepharose 4B anti-human IgG column.

*NCS conjugation to MAb A7 and chA7Fab*

MAb A7 was conjugated to NCS with SPDP as described previously (Kimura *et al.*, 1980). The conjugation ratio used was 2 mol of NCS per mol of MAb A7, i.e. 7.5 mg of MAb A7 was bound to 1 mg of NCS. ChA7Fab was conjugated to NCS as described previously (Yamaguchi *et al.*, 1993). The conjugation ratio used was 1 mol of NCS per mol of chA7Fab, i.e. 4.5 mg of chA7Fab was bound to 1 mg of NCS.

In vivo *effects of A7-NCS on HPC-YS tumours*

Anti-tumour effects of chA7Fab-NCS were investigated using nude mice bearing HPC-YS tumours that are known to react with chA7Fab. Small tumours were detected in all mice 14 days after inoculation; they were then divided into five groups: six mice received chA7Fab-NCS conjugate in PBS (chA7Fab, 2.25 mg $kg^{-1}$; NCS, 500 $\mu g\ kg^{-1}$), six received A7-NCS conjugate (A7, 3.75 mg $kg^{-1}$; NCS, 500 $\mu g\ kg^{-1}$), six received NCS in PBS (500 $\mu g\ kg^{-1}$), six received chA7Fab in PBS (2.25 mg $kg^{-1}$), and six received PBS as a control. The mean tumour size was similar between different treatment groups. All treatments were given by intravenous injection into the tail vein in 100 $\mu l$ of PBS. The drug solution was administered daily for 5 days. Tumours were measured (maximum length and width) twice a week until day 31. Tumour volume ($V$) was calculated using the formula, $V = (a^2 \times b)/2$, where $a$ was the maximum width and $b$ was the maximum length. To standardise any variation in tumour volume, the relative tumour volume (RV) was calculated using the formula, $RV = V_2/V_1$, where $V_1$ was the initial tumour volume and $V_2$ was the tumour volume at any given time. The data are presented as the mean $\pm$ standard error (s.e.). In other experiments, the anti-tumour effects of twice the concentration of chA7Fab-NCS, A7-NCS and NCS were tested. Student's $t$-test was used to determine statistically significant differences. A $P$-value $< 0.05$ was considered significant.

In vivo *effects of A7-NCS on KB-2 tumours*

Anti-tumour effects of chA7Fab-NCS on KB-2 tumours, which do not react with chA7Fab, were investigated. The chA7Fab conjugate, free NCS solution and PBS were administered as above, and the size of the tumours was measured on the same schedule as above.

*Antigenicity of MAb A7 and murine and chimeric Fab fragments of MAb A7*

An enzyme-linked immunosorbent assay (ELISA) was performed using MAb A7 and murine and chimeric Fab fragments of MAb A7. Various concentrations of MAb A7 and murine and chimeric Fab fragments of MAb A7 in carbonate-bicarbonate buffer, pH 8.4, were added to 96-well microtitre plates (Flow Laboratories, MD, USA) and incubated at 37°C for 2 h. After five washes with 0.05% Tween 20 in PBS, the wells were treated with 25% Block Ace (Dainippon Kagaku, Tokyo, Japan) in PBS. Following removal of Block Ace, the wells were incubated with 100 $\mu l$ of peroxidase-labelled rabbit anti-mouse IgG antibody (Zymed Laboratories, CA, USA) for 30 min. After five rinses with 0.05% Tween 20 in PBS, 100 $\mu l$ of the substrate solution, consisting of 0.1 M citrate buffer, pH 4.0, containing azinodiethylbenzolin (ABTS) (0.5 mg $ml^{-1}$) and 0.01% hydrogen peroxide, was added to each well. The absorbance was read at 414 nm by an automatic immunoreader (Bio-Rad, CA, USA).

### Results

In vivo *anti-tumour effects of A7-NCS on HPC-YS tumours*

Tumour volumes on day 31 were larger than at the beginning of treatment (day 14) in all mice. The relative tumour volume

**Figure 1** HPC-YS tumour growth curves in mice receiving intravenous chA7Fab-NCS conjugate (chA7Fab, 2.25 mg $kg^{-1}$; NCS, 500 $\mu g\ kg^{-1}$), A7-NCS conjugate (A7, 3.75 mg $kg^{-1}$; NCS, 500 $\mu g\ kg^{-1}$), free NCS (500 $\mu g\ kg^{-1}$), chA7Fab (2.25 mg $kg^{-1}$), or PBS. A statistically significant difference was observed in the RV on day 31 between mice administered NCS solution, only chA7Fab or PBS and those receiving chA7Fab-NCS or A7-NCS ($P < 0.01$). ○, chA7Fab-NCS; △, A7-NCS; □, free NCS; ◇, chA7Fab; X, PBS; points, means; bars, s.e.; *, significant difference ($P < 0.01$).

**Figure 2** HPC-YS tumour growth curves in mice receiving intravenous chA7Fab-NCS conjugate (chA7Fab, 4.5 mg kg$^{-1}$; NCS, 1000 $\mu$g kg$^{-1}$), A7-NCS conjugate (A7, 7.5 mg kg$^{-1}$; NCS, 1000 $\mu$g kg$^{-1}$), or free NCS (1000 $\mu$g kg$^{-1}$). Tumour growth was completely suppressed only in the chA7Fab-NCS treated group. A statistically significant difference was observed in the RV on day 31 between mice administered chA7Fab-NCS and all other groups ($P$<0.01). ○, chA7Fab-NCS; △, A7-NCS; □, free NCS; X, PBS; points, means; bars, s.e.; *, significant difference ($P$<0.01).

**Figure 3** KB-2 tumour growth curves in mice receiving intravenous chA7Fab-NCS conjugate (chA7Fab, 2.25 mg kg$^{-1}$; NCS, 500 $\mu$g kg$^{-1}$), A7-NCS conjugate (A7, 3.75 mg kg$^{-1}$; NCS, 500 $\mu$g kg$^{-1}$), free NCS (500 $\mu$g kg$^{-1}$), or PBS. ○, chA7Fab-NCS; △, A7-NCS; □, free NCS; X, PBS; points, means; bars, s.e.

(RV) in the control mice administered PBS was 6.92±0.38 on day 31. The RV in mice administered chA7Fab only was 6.28±0.38 on day 31. By contrast, the RV of mice treated with the chA7Fab-NCS conjugate was 1.87±0.08 while the RV was 2.78±0.10 and 4.21±0.12 for mice treated with A7-NCS and NCS alone, respectively. A statistically significant difference was observed in the RV on day 31 between mice given NCS solution, chA7Fab alone or PBS and those receiving chA7Fab-NCS or A7-NCS ($P$<0.01), but not between the groups treated with chA7Fab-NCS and A7-NCS (Figure 1).

Figure 2 shows the results for the mice which received twice the drug doses (chA7Fab, 4.5 mg kg$^{-1}$; NCS, 1000 $\mu$g kg$^{-1}$), A7-NCS (A7, 7.5 mg kg$^{-1}$; NCS, 1000 $\mu$g kg$^{-1}$), free NCS (1000 $\mu$g kg$^{-1}$) and PBS. The day 31 RV in these mice were 0.89±0.29, 2.03±0.30, 3.2±0.19 and 7.1±0.46 for the chA7Fab-NCS, A7-NCS, free NCS and PBS-treated groups, respectively. Tumour growth was completely suppressed only in the chA7Fab-NCS-treated group, and this difference was statistically significant compared with the A7-NCS, NCS and PBS groups ($P$<0.01). Although a statistically significant difference was not observed between the mice treated with A7-NCS and NCS, the tumours in the former group tended to be smaller.

### In vivo *anti-tumour effect of A7-NCS on KB-2 tumours*

The effects of chA7Fab-NCS, A7-NCS and free NCS on KB-2 tumours were nearly identical at both doses tested (Figures 3 and 4).

### *Antigenicity of MAb A7, and murine and chimeric Fab fragments of MAb A7*

The chA7Fab-NCS conjugate reacted the least with rabbit anti-mouse IgG. In contrast, MAb A7 reacted very strongly in the ELISA (Figure 5).

### Discussion

MAb conjugates with chemotherapeutic drugs may improve the therapeutic efficacy of these drugs by increasing the

**Figure 4** KB-2 tumour growth curves in mice receiving intravenous chA7Fab-NCS conjugate (chA7Fab, 4.5 mg kg$^{-1}$; NCS, 1000 $\mu$g kg$^{-1}$), A7-NCS conjugate (A7, 7.5 mg kg$^{-1}$; NCS, 1000 $\mu$g kg$^{-1}$), or free NCS (1000 $\mu$g kg$^{-1}$). ○, chA7Fab-NCS; △, A7-NCS; □, free NCS; points, means; bars, s.e.

**Figure 5** ELISA for the antigenicity of the chA7Fab, murine Fab fragment of MAb A7, and whole MAb A7 using rabbit anti-mouse immunoglobulin. ○, chA7Fab; △, murine Fab fragments of MAb A7; X, MAb A7; points, means; bars, s.d.

localisation of the drug to the tumour and by minimising their toxic effects; the latter is a major limitation in conventional chemotherapy. We have conjugated NCS to MAb A7, which has been used to treat over 80 patients with colorectal carcinoma. Some of these patients have had a substantial regression of their tumour (Takahashi *et al.*, 1993).

We chose the anti-tumour drug NCS because NCS can be covalently joined to an antibody without destroying its anti-tumour activity (Jung *et al.*, 1981). NCS has been reported to contain a non-protein chromophore that is not covalently bound to the protein and is the active part of NCS (Napier *et al.*, 1989; Ohtsuki and Ishida, 1980). Because MAb A7 reacted with 77% of human pancreatic carcinoma cell lines (Otsuji, *et al.*, 1993*a*), we investigated the applicability of MAb A7 for targeting NCS in pancreatic cancer. In general, the variable fragment of MAbs has the ability to leave the vascular space rapidly and to penetrate target tumour tissue (Sutherland *et al.*, 1987). Thus, the Fab fragment of MAb A7 may be able to carry a large amount of a short-acting anti-cancer drug like NCS directly into the tumour. In a study (Otsuji *et al.*, 1994*a*) using pancreatic cancer xenografts in a nude mouse model, $^{125}$I-labelled chA7Fab-NCS accumulated more rapidly and in significantly larger amounts 1 h after injection, a time when NCS is still active. Moreover, because chA7Fab-NCS is catabolised more rapidly than A7-NCS, thus accelerating the clearance of NCS, chA7Fab-NCS can be administered more often. In this study, the *in vivo* anti-tumour effect of chA7Fab-NCS on HPC-YS tumours was greater than that of A7-NCS and only chA7Fab-NCS (chA7Fab, 4.5 mg kg$^{-1}$; NCS, 1000 $\mu$g kg$^{-1}$) completely suppressed tumour growth.

When mice bearing KB-2 tumours, to which MAb A7 does not bind, were studied, an enhanced cytostatic effect of chA7Fab-NCS was not observed and there was no significant difference between the effects of chA7Fab-NCS and the other NCS groups. These results suggest that the increased anti-tumour effect of chA7Fab-NCS on HPC-YS was caused by specific antigen-antibody binding.

Murine MAbs administered to humans induce a HAMA response (Frodin *et al.*, 1986; Lobugli *et al.*, 1986; Blottiere *et al.*, 1987) that may reduce tumour localisation. Takahashi *et al.* (1993) reported previously that HAMA was produced in all patients who received A7-NCS. Cyclosporin has been used to suppress the formation of HAMA as reported by Weiden *et al.* (1994). However, adverse effects such as elevation of bilirubin and creatinine and increases in blood pressure can occur with administration of cyclosporin. Although some groups have developed methods to produce human antibodies and reported some promising data (Hoogenboom and Winter, 1992), they are not widely used clinically. To reduce HAMA responses potentially, we produced chA7Fab by recombinant DNA techniques. Because of the human origin of the shortened Fc portion of the chimeric Fab fragment of MAb A7, HAMA production should be decreased when this conjugate is administered to humans. We could not evaluate any differences in the antigenicity of intact MAb A7 and the chimeric Fab fragment of MAb A7 in humans because we have not yet performed *in vivo* human experiments. However, in an immunological study using rabbit anti-mouse antibody, the antigenicity of the chA7Fab was lower than the intact MAb A7 or murine Fab fragments of MAb A7. Although this experiment was performed using an *in vitro* model and this situation was somewhat different from the clinical setting, these results suggest that chA7Fab may be less antigenic to humans than the intact MAb or the murine Fab fragment of MAb A7.

We conclude that chA7Fab-NCS can inhibit human pancreatic cancer growth in an animal xenograft and that chA7Fab may be useful for targeting chemotherapy to pancreatic cancer patients.

**Acknowledgements**
This work was supported in part by a Grant-in-Aid from the Ministry of Health and Welfare and by a Grant-in-Aid from the Pancreatic Research Foundation of Japan

### References

APELGREN LD, ZIMMERMAN DL, BRIGGS SL AND BUMOL TF. (1990). Antitumor activity of monoclonal antibody - Vinca alkaloid immunoconjugate LY203725 (KS1/4-4-desacetylvinblastine-3-carboxhydrazide) in a nude mouse model of human ovarian cancer. *Cancer Res.*, **50**, 3540-3544.

BLOTTIERE HM, MAUREL C AND DOUILLARD JY. (1987). Immune function of patients with gastrointestinal carcinoma after treatment with multiple infusions of monoclonal antibody 17.1A. *Cancer Res.*, **47**, 5238-5241.

BOULIANNE GL, HOZUMI N AND SCHULMAN MJ. (1984). Production of functional chimeric mouse/human antibody. *Nature*, **312**, 643-646.

DEGUCHI T, MING C, SUSAN SL, JURIUS SH AND LEE C. (1987). Potential therapeutic effect of adriamycin-monoclonal anti-prostatic acid phosphatase antibody conjugate on human prostate tumor. *J. Urol.*, **137**, 353-358.

FRODIN JE, BIBERFELD P, CHRISTENSSON B, PHILSTEDT P, SUDELIUS S, SYLVEN M, WAHREN B, KOPROWSKI H AND MELLSTEDT H. (1986). Treatment of patients with metastasizing colo-rectal carcinoma with mouse monoclonal antibodies: a progress report. *Hybridoma*, **5**, S151-S161.

FUJITA H, NAKAYAMA N, SAWABE T AND KIMURA K. (1970). *In vivo* distribution and inactivation of neocarzinostatin. *Jpn. J. Antibiotics*, **23**, 471-478.

FUKUDA K. (1985). The study of targeting chemotherapy against gastrointestinal cancer. *Akita J. Med.*, **12**, 451-468.

HASPEL MV, MCCABE RP, POMATO N, JANESCH NJ, KNOWLTON JV, PETERS LC, HOOVER HC JR AND HANNA MG JR. (1985). Generation of tumor cell-reactive human monoclonal antibodies using peripheral blood lymphocytes from actively immunized colorectal patients. *Cancer Res.*, **45**, 3951-3961.

HOOGENBOOM HR AND WINTER G. (1992). By-passing immunisation. Human antibodies from synthetic repertories of germline VH gene segments rearranged *in vitro*. *J. Mol. Biol.*, **20,** 381-388.
JUNG G, KOHNLEIN W AND LUDERS G (1981). Biological activity of the antitumor protein neocarzinostatin coupled to a monoclonal antibody by *N*-succinimidyl 3-(2-pyridyldithio)-propionate. *Biochem. Biophys. Res. Commun.*, **101,** 599-606.
KIMURA I, OHNISHI T, TSUBOTA T, SATO Y, KOBAYASHI T AND ABE S. (1980). Production of tumor antibody-neocarzinostatin conjugate and its biological activities. *Cancer Immunol. Immunother.*, **7,** 235-242.
LOBUGLI AF, SALEH M, PETERSON L, WHEELER R, CARRANO R, HUSTER W AND KHAZAELI MB. (1986). Phase 1 clinical trial of CO17-1A monoclonal antibody. *Hybridoma*, **5,** S117-S123.
MCCABE RP, PETERS LC, HASPEL MV, POMATO N, CARRASQUILLO JA AND HANNA MG JR. (1988). Preclinical studies on the pharmacokinetic properties of human monoclonal antibodies to colorectal cancer and their use for detection of tumors. *Cancer Res.*, **48,** 4348-4353.
MORRISON SL, JOHNSON MJ, HERZENBERG LA AND OI VT. (1984). Chimeric human antibody molecules: mouse antigen-binding domains with human constant region domains. *Proc. Natl Acad. Sci. USA*, **81,** 6851-6855.
NAPIER MA, KAPPEN LS AND GOLDBERG IH. (1989). Effect of nonprotein chromophore removal on neocarzinostatin action. *Biochemistry*, **19,** 1767-1773.
OHTSUKI K AND ISHIDA N. (1980). The biological effect of a nonprotein component removed from neocarzinostatin (NCS). *J. Antibiot.*, **33,** 744-750.
OHYANAGI H, ISHIDA H, ISHIDA T, SOYAMA N, YAMAMOTO M, OKUMURA S, KANO Y, UEDA Y AND SAITOH Y. (1988). A monoclonal antibody, KM10, reactive with human gastrointestinal cancer and its application for immunotherapy. *Jpn. J. Cancer Res.*, **79,** 1349-1358.
OKABAYASHI K, NAKAGAWA Y, HAYASUKE N, OHI H, MIURA M, ISHIDA Y, SHIMAZU M, MURAKAMI K, HIRABAYASHI K, MINATOMI H, HORII H, MASAKI A, SUMI A, OHMURA T AND KAWABE H. (1991). Secretory expression of the human serum albumin gene in the yeast, *Saccharomyces cerevisiae*. *J. Biochem.*, **110,** 103-110.
OTSUJI E, TAKAHASHI T, YAMAGUCHI T, YAMAGUCHI N AND IMANISHI J. (1990). Specific cytotoxic effect of neocarzinostatin conjugated to monoclonal antibody A7 on human pancreatic carcinoma. *Gastroenterol. Jpn.*, **25,** 244-248.
OTSUJI E, YAMAGUCHI T, YAMAGUCHI N, KOYAMA K, IMANISHI J, YAMAOKA N AND TAKAHASHI T. (1992). Expression of the cell surface antigen detected by the monoclonal antibody A7 in pancreatic carcinoma cell lines. *Surg. Today*, **22,** 351-356.
OTSUJI E, YAMAGUCHI T, YAMAOKA N, KITAMURA K, YAMAGUCHI N, IMANISHI J AND TAKAHASHI T. (1993*a*). Increased antitumor effect of neocarzinostatin conjugated to monoclonal antibody A7 on human pancreatic carcinoma grafted in nude mice. *Antibody, Immunoconj. Radiopharm.*, **6,** 177-183.
OTSUJI E, YAMAGUCHI T, YAMAOKA N, KATO M, KOTANI T, KITAMURA K, YAMAGUCHI N AND TAKAHASHI T. (1993*b*). Enhanced tumor localization of radiolabeled Fab fragments of monoclonal antibody A7 in nude mice bearing human pancreatic carcinoma xenografts. *Jpn. J. Cancer Res.*, **84,** 914-920.
OTSUJI E, YAMAGUCHI T, YAMAOKA N, TANIGUCHI K, KATO M, KOTANI T, KITAMURA K AND TAKAHASHI T. (1994*a*). Biodistribution of neocarzinostatin conjugated to chimeric Fab fragments of the monoclonal antibody A7 in nude mice bearing human pancreatic cancer xenografts. *Jpn. J. Cancer Res.*, **85,** 530-535.
OTSUJI E, YAMAGUCHI T, YATA Y, NISHI H, OKAMOTO K, TANIGUCHI K, KATO M, KOTANI T, YAMAOKA N, KITAMURA K AND TAKAHASHI T. (1994*b*). Antitumor effect of neocarzinostatin conjugated to human/mouse chimeric Fab fragments of the monoclonal antibody A7 on human pancreatic carcinoma. *J. Surg. Oncol.*, **57,** 230-234.
SPIEGELBERG HL AND WEIGLE WO. (1965). The catabolism of homologous and heterologous 7s gamma globulin fragments. *J. Exp. Med.*, **121,** 323-338.
SUTHERLAND R, BUCHEGGER F, SCHREYER M, VACCA A AND MACH JP. (1987). Penetration and binding of radiolabeled anti-carcinoembryonic antigen monoclonal antibodies and their antigen binding fragments in human colon multicellular tumor spheroids. *Cancer Res.*, **47,** 1627-1633.
TAKAHASHI T, YAMAGUCHI T, KITAMURA K, NOGUCHI A, HONDA M AND OTSUJI E. (1993). Follow-up study of patients treated with monoclonal antibody-drug conjugate: report of 77 cases with colorectal cancer. *Jpn. J. Cancer Res.*, **84,** 976-981.
WEIDEN PL, WOLF SB, BREITZ HB, APPELBAUM JW, SEILER CA, MALLETT R, BJORN MJ, SU FM, FER MF AND SALK D. (1994). Human anti-mouse antibody suppression with cyclosporin A. *Cancer*, **73,** 1093-1097.
YAMAGUCHI T, TSURUMI H, KITAMURA K, OTSUJI E, MIYAGAKI T, KOTANI T AND TAKAHASHI T. (1993). Production, binding and cytotoxicity of human/mouse chimeric monoclonal antibody-neocarzinostatin conjugate. *Jpn. J. Cancer Res.*, **84,** 1190-1194.